# Emerging role of the endoplasmic reticulum in peroxisome biogenesis

**DOI:** 10.3389/fphys.2013.00286

**Published:** 2013-10-08

**Authors:** Gaurav Agrawal, Suresh Subramani

**Affiliations:** Section of Molecular Biology, Division of Biological Sciences, University of CaliforniaSan Diego, La Jolla, CA, USA

**Keywords:** peroxisome, intracellular protein trafficking, organelle biogenesis, ER involvement in peroxisome biogenesis, vesicle budding, peroxisomal ER

## Abstract

During the past few years, we have witnessed a paradigm shift in our long-standing concept of peroxisome biogenesis. Recent biochemical and morphological studies have revealed a primary role of the endoplasmic reticulum (ER) in the *de novo* formation of peroxisomes, thus challenging the prevalent model invoking growth and division of pre-existing peroxisomes. Importantly, a novel sorting process has been recently defined at the ER that segregates and assembles specific sets of peroxisomal membrane proteins (PMPs) into distinct pre-peroxisomal vesicular carriers (ppVs) that later undergo heterotypic fusion to form mature peroxisomes. Consequently, the emerging model has redefined the function of many peroxins (most notably Pex3, Pex19, and Pex25) and assigned them novel roles in vesicular budding and subsequent peroxisome assembly. These advances establish a novel intracellular membrane trafficking route between the ER and peroxisomes, but the components remain elusive. This review will provide a historical perspective and focus on recent developments in the emerging role of the ER in peroxisome biogenesis.

## Introduction

The peroxisome, along with glycosomes and glyoxysomes, is a member of the microbody family of subcellular organelles. Peroxisomes are ubiquitously present in all eukaryotes. Their primary function is to sequester several metabolic enzymes that are involved in the β-oxidation of long-chain fatty acids, formation of bile acids, dolichol, and cholesterol (Van Veldhoven, 2010). In methylotropic yeasts, peroxisomes are essential for the metabolism of methanol (Subramani, 1998). In plants, peroxisomes house the glyoxylate cycle enzymes and also participate in photorespiration. In humans and other mammals, peroxisomes are required for the synthesis of plasmalogens that are vital membrane components of the heart and brain (Brites et al., 2009). Peroxisomes share several steps of these metabolic pathways with mitochondria, chloroplasts, ER, and cytosol through redox shuttles.

## Background

Early enzyme distribution studies led to the discovery of peroxisomes. In early 1950s, cellular fractionation of tubular cells of mouse kidney and liver parenchymal cells revealed dense, single membrane cytoplasmic bodies with a granular matrix (Rhodin, 1954; Bernhard and Rouiller, 1956). These structures were introduced in the electron microscopy literature as “microbodies.” Toward the early 1960s, an extensive characterization for the enzymatic content of microbodies revealed the abundance of a variety of oxidases and catalase, among other enzymes. The association of these enzymes in a single organelle was biologically meaningful, particularly for the disposal of hydrogen peroxide, which is highly injurious to cell components. Thus, the segregation of the enzymes producing hydrogen peroxide together with an enzyme, catalase that effectively metabolizes it could be viewed as having an essentially protective function. Hence the term “peroxisome” was proposed (de Duve, 1960, 1965). However, since the discovery of peroxisomes, their cellular origin has been actively debated.

The prevailing view of the cellular origin of peroxisomes has been evolving and has narrowed to two alternative routes, one apparently more prevalent in mammalian cells (growth and division model) and the other in yeasts and plants [*de novo* model involving the endoplasmic reticulum (ER)]. However, this review will focus on how the field is converging toward a more generalized paradigm for mammalian and yeast systems, where both routes could be operating simultaneously. If true, understanding the environmental cues that shift this balance in favor of one model over the other will be very important. As mentioned above, the initial biochemical characterization has revealed a strong metabolic role of peroxisomes. This lead de Duve and others to suggest that peroxisomes, like mitochondria and chloroplasts, could be an autonomous organelle and endosymbiont in origin and multiply through growth and division of pre-existing organelles (de Duve, 1982; Lazarow and Fujiki, 1985). However, an ER origin of peroxisomes was proposed based on parallel observations of Novikoff and colleagues, that the peroxisomal membrane has continuities with the smooth ER and could be conceived as buds forming at the terminal ends of certain specialized areas of the ER (Rhodin, 1954). They observed that a stalk-like structure attaches the peroxisomes to these ER regions that could eventually bud off into the cytoplasm.

## Support for the growth and division model as an exclusive route for peroxisome biogenesis

In the 1980s, Lazarow and colleagues (Rachubinski et al., 1984; Lazarow and Fujiki, 1985; Lazarow, 1989) made observations that supported the growth and division model of peroxisome biogenesis in mammalian cells. Their key observations were, firstly, that matrix and membrane peroxins were synthesized on free ribosomes in the cytosol and were then sorted to pre-existing peroxisomes. Secondly, in mutants in which matrix protein import is impaired, membrane “ghosts” or peroxisomes remnants were present and these could provide a structural scaffold to reassemble functional peroxisomes once the missing peroxin is reintroduced by genetic complementation (Santos et al., 1988). Supporting this view was the observation that vesicles and tubular structures, possibly corresponding to peroxisome remnants, were observed with deconvolution microscopy in *pex3*Δ cells of *P. pastoris* (Hazra et al., 2002). Similarly, in *H. polymorpha*, vesicular membrane structures formed by the expression of a 50aa N-terminal fragment of Pex3 could act as precursors for reforming normal peroxisomes, when cells were complemented with the full-length Pex3 construct (Faber et al., 2002). It was noted that there are no peroxisomal remnants or “ghosts” in mutants in which the genes for two or more essential peroxins are deleted (e.g., *pex3*Δ, *pex16*Δ, *pex19*Δ). A major flaw in the hypothesis that growth and division is the only process for peroxisome biogenesis is that these cells are capable of regenerating peroxisomes when the missing genes are reintroduced, despite the observation that pre-existing peroxisomes are undetectable in the mutant cells.

Arguments against the *de novo* peroxisome biogenesis and the involvement of the ER came from the following negative observations. Firstly, inhibitors of COPI and COPII vesicle formation failed to inhibit peroxisome biogenesis in mammalian cells (South et al., 2000; Voorn-Brouwer et al., 2001). Secondly, in mutants with an inactive Sec61, a protein that forms the ER translocon essential for the entry of proteins into the ER, peroxisome biogenesis was unaffected (South et al., 2001). However, in hindsight these observations only suggested that the components of the standard secretory pathway were not required for peroxisome biogenesis. Lastly, if peroxisomes were to be formed from the ER, peroxins like Pex3, Pex16, and Pex19 that are essential for the assembly of peroxisomal membrane proteins (PMPs) should be localized to the ER (Lazarow, 2003). However, the majority of researchers never found Pex3, Pex16, and Pex19 or other PMPs localized to the ER in mammalian cells, even when peroxisomes are absent, perhaps due to the instability or aggregation of these PMPs in mutant cells (Voorn-Brouwer et al., 2001; Fang et al., 2004; Hunt and Trelease, 2004). In contrast, recent reports have emerged that show a transient localization of certain PMPs to the ER (Geuze et al., 2003; Kim et al., 2006; Yonekawa et al., 2011) and there is clear evidence in yeast that many PMPs do transit to peroxisomes via the ER (Titorenko and Rachubinski, 1998; Hoepfner et al., 2005; Yan et al., 2008; Agrawal et al., 2011; Joshi et al., 2012).

The growth and division model involves Pex19 and Pex3 in performing the post-translational insertion of PMPs into the peroxisomal membrane (Jones et al., 2004; Matsuzono and Fujiki, 2006; Matsuzaki and Fujiki, 2008). Earlier, using pulse-chase experiments, PMP70 was chased from the cytosol to mammalian peroxisomes without transiting any ER-like compartment (Imanaka et al., 1996). Similarly, another mammalian protein, PMP22, was post-translationally incorporated *in vitro* into purified peroxisomes (Diestelkotter, 1993). PMPs that depend on Pex19 for their targeting to the peroxisomal membrane are classified as Type I PMPs, whereas those that do not require Pex19 are termed as Type II PMPs (Jones et al., 2004). Except a few PMPs (Pex3 and Pex22), all other studied are either Type I or tail-anchored (TA) PMPs. In mammalian cells, Pex19 binds and stabilizes newly synthesized PMPs through their hydrophobic domains in the cytoplasm and acts as a chaperone and an import receptor to insert them into the peroxisomal membrane. The translated PMPs, which are soluble when Pex19 is present, form aggregates in its absence (Shibata et al., 2004; Kashiwayama et al., 2005). Pex19 binds to specific “*cis-acting*” peroxisome targeting signals within PMPs called mPTSs (Jones et al., 2001, 2004; Rottensteiner et al., 2003), which are important for their targeting to the peroxisomal membrane. PMPs are mislocalized either when their mPTSs are mutated or when Pex19 is missing, as in *pex19*Δ cells (Sacksteder et al., 2000; Jones et al., 2004; Halbach et al., 2009). After Pex19 has bound the mPTS domain/s of PMPs, it binds to Pex3 present on the peroxisomal membrane, thereby inserting the bound PMP into the membrane. For accomplishing these tasks, Pex19 uses non-overlapping binding sites that recognize Pex3 and mPTSs (Fransen et al., 2005; Sato et al., 2010; Schueller et al., 2010). In addition, Pex19 has been also shown to incorporate TA proteins directly into the peroxisome membrane (Fujiki et al., 2006; Matsuzono and Fujiki, 2006; Halbach et al., 2009) independent of the classical TRC40 pathway (Yagita et al., 2013). Otherwise, the TRC40/GET pathway is widely accepted as the dominant pathway for targeting and inserting TA proteins into cellular membranes, including the ER (Borgese and Fasana, 2011). Upon accomplishing membrane biogenesis, peroxisomes acquire import competence for matrix enzymes and eventually grow and will undergo division to meet the metabolic requirements of the cell. The growth and division model side-steps the issue of where membrane lipids are derived from for peroxisome growth and how they are inserted into the membrane. However, very different roles of Pex19 and Pex3 are proposed in the *de novo* peroxisome biogenesis model (see below).

## The ER as a precursor for PMP biogenesis

Though there were intermittent reports (Gonzalez and Beevers, 1976; Ohno and Fujii, 1990), renewed focus on the ER did not occur until the late 1990s. In *S. cerevisiae*, Pex15, a TA protein, was suggested to traffic from the ER to the peroxisomes (Elgersma et al., 1997). An ER targeting signal overlapping with its mPTS was found in Pex15, and its overexpression caused profound proliferation of the ER membrane. In the following year, another study, this time in *Y. lipolytica* showed a more direct involvement of the ER in peroxisome biogenesis. The temperature-sensitive mutants of *SEC238* and *SRP54*, whose genes products are involved in the secretory pathway, not only inhibited the exit of an alkaline extracellular protease from the ER, but also lead to temperature-sensitive growth of cells in peroxisome proliferating conditions (Titorenko and Rachubinski, 1998, 2000). In addition, this study also showed two other peroxins, Pex2, and Pex16, which were delivered to the peroxisomes via the ER. The two peroxins were pulse-labeled and were imported from the cytosol to the ER, N-glycosylated in the ER-lumen and then chased to the peroxisomes. Unlike some of the previous studies (Baerends et al., 1996; Komori et al., 1997; Kammerer et al., 1998), these observations were more relevant physiologically since the PMPs were not overexpressed. Additional reports for the involvement of the ER came from yeast and plant cells treated with Brefeldin A (BFA; a fungal toxin that inhibits vesicle transport from the ER). In *H. polymorpha* cells treated with BFA, several peroxins accumulated in a structure resembling the ER (Salomons et al., 1997). In plants, a peroxisomal isoform of ascorbate peroxidase (APX) was localized to the reticular ER, in addition to the peroxisomes (Mullen et al., 1999). But treatment with BFA restricted the localization of APX to the ER-like structures and this could be reversed by removal of BFA. These ER-like structures lacked typical ER resident proteins, such as BiP2, calnexin, and calreticulin. In addition, the *in vitro* translated APX could only be incorporated into the ER membranes and not into any other organelle membranes (including peroxisomal membranes), suggesting that the ER hosts the protein before it is trafficked to the peroxisomes. In addition, other studies identified several ER-associated proteins of the secretory pathway that are necessary for peroxisome assembly. Previously the *SEC238* and *SRP54* genes in *Y. lipolytica* were found to be essential for the exit of Pex2 and Pex16 from the ER and for peroxisome assembly (Titorenko and Rachubinski, 1998). More recently, it was reported that repression of other ER proteins, Sec20, Sec39, and Dsl1, causes the mislocalization of Pex3 and Pot1 (Perry et al., 2009). Collectively, these studies provide compelling evidence for the ER as the precursor for peroxisomes, at least in yeast and plants. These studies were viewed with skepticism initially because they were counter to the widely accepted growth and division model (Lazarow, 2003) and negative experiments ruling out a role for certain components of the ER-secretory pathway (South and Gould, 1999; Voorn-Brouwer et al., 2001). However, this view has shifted over the past decade.

## *De novo* peroxisome biogenesis in yeast

The reappearance of peroxisomes in cells completely lacking pre-existing peroxisomes presented the most relevant argument against the growth and division model. During the discovery of the genes essential for peroxisome biogenesis, mainly *PEX3* and *PEX19*, the reintroduction of a functional copy of these genes restored peroxisome biogenesis in the mutant cells (Hohfeld et al., 1991; Erdmann and Kunau, 1992; Baerends et al., 1996; Wiemer et al., 1996). However, this was not the case with mitochondrial biogenesis mutants, where reintroduction of the corresponding functional gene could not rescue the organelle (Ryan and Hoogenraad, 2007). Later in 2005, Tabak and colleagues (Hoepfner et al., 2005) provided more conclusive proof for the involvement of ER in peroxisome biogenesis. They followed the intracellular route for the newly-synthesized, YFP-tagged Pex3 and Pex19 in *pex3*Δ, *pex19*Δ, and wild-type cells using advanced real-time fluorescence microscopy and biochemical experimentation. Pex3 first appears at the perinuclear ER and can be followed to punctate structures coinciding with the ER. These dot-like structures later detach in a Pex19-dependent manner from the ER and start to co-localize with matrix proteins representing import-competent peroxisomes. In addition, Pex3 remains trapped in the ER in the absence of Pex19; and without Pex3, Pex19 never localizes to the ER membrane (Hoepfner et al., 2005). These data suggest that metabolically-active peroxisomes are formed *de novo* from the ER through the recruitment of Pex19 by ER-localized Pex3.

**Figure 1:**
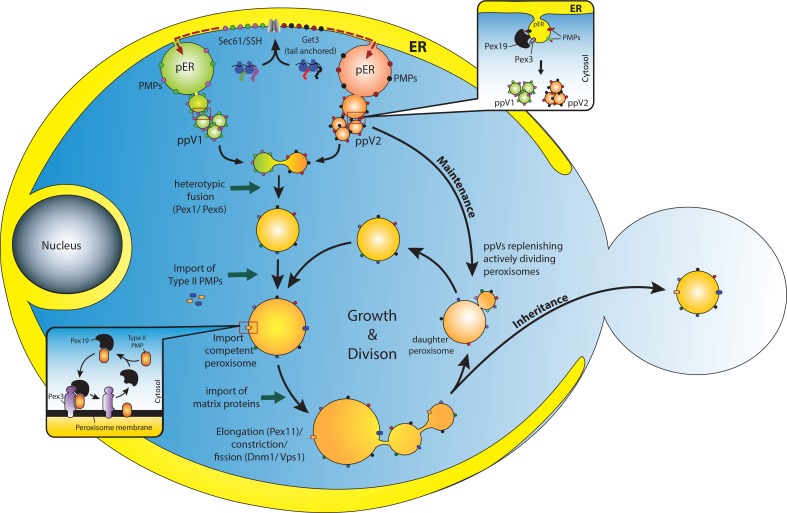
**Overview of peroxisome biogenesis: converging *de novo* and growth and division models**. PMPs are translated in the cytosol on free ribosomes and are incorporated into the ER membrane through specific translocons (Sec61 or Get3) (van der Zand et al., 2010; Borgese and Fasana, 2011). It is assumed that the PMPs are segregated and sorted (presumably, through an intramolecular signal) into at least two different pre-peroxisomal compartments (pER) in the ER (Fakieh et al., 2013). These PMPs leave the ER through vesicular carriers that bud in a Pex19 dependent manner (upper inset) (Lam et al., 2010; Agrawal et al., 2011). These vesicles undergo heterotypic fusion to unite the components of the importomer to form import competent peroxisomes (van der Zand et al., 2012), a process that requires the Pex1 and Pex6 proteins (Titorenko et al., 2000). It is expected that Type II PMPs are incorporated at this stage since they are essential for the import of matrix proteins (Koller et al., 1999). Here Pex19 might act as an mPTS receptor that binds and stabilizes the Type II PMPs in the cytosol and incorporates them into the peroxisomal membrane upon docking to the membrane bound Pex3 (Schmidt et al., 2012) (lower inset). Upon subsequent import of matrix proteins, peroxisomes grow in size and undergo division to replenish cells with an adequate number of peroxisomes to meet metabolic requirements of the cells. This pathway could be the most plausible way by which peroxisomes are repopulated in cells lacking peroxisomes (such as *pex3*Δ/ *pex19*Δ) when the missing gene is reintroduced (Hoepfner et al., 2005; van der Zand et al., 2010; Agrawal et al., 2011). However, in WT cells as well, ER might supply PMPs to replenish and maintain the actively dividing peroxisomes (Motley and Hettema, 2007). An active interaction between the two pathways would enable cells to adapt dynamically to changing environments.

Subsequent studies established PMP traffic to peroxisomes via the ER as a rule, rather than as an exception, not only in *S. cerevisiae*, but also in other yeasts (Kragt et al., 2005; Tam et al., 2005; Yan et al., 2008; Knoblach and Rachubinski, 2010; van der Zand et al., 2010; Huber et al., 2011; Saraya et al., 2011; Joshi et al., 2012) (Figure 1). At least 20 different PMPs have been followed from the ER to the peroxisomes irrespective of their membrane topologies or function in peroxisome biogenesis. Interestingly, van der Zand et al. found that the ER-routed trafficking of more than 15 different PMPs was not restricted only during the *de novo* formation of peroxisomes, but was detected in wild-type cells already containing peroxisomes (van der Zand et al., 2010). The authors thus suggest a unified PMP biogenesis route in both wild-type and mutant cells. However, Motley and Hettema suggested that peroxisomes are formed *de novo* only when pre-existing peroxisomes are absent, as seen in cells with a defect in peroxisome inheritance, whereas growth and division is the default pathway for peroxisome biogenesis in wild-type cells (Motley and Hettema, 2007). They also showed that newly formed Pex3-GFP is trafficked through ER in both wild-type and *pex3*Δ *cells*, but is targeted to the pre-existing peroxisomes in the wild-type cells (since they do not detect *de novo* formation of peroxisomes in wild-type cells), but localizes to new peroxisomes in *pex3*Δ cells. This extended the role of the ER from *de novo* peroxisome biogenesis to the maintenance of the pre-existing peroxisomes population (for renewing them with fresh PMPs). This would also explain how the pre-existing peroxisomes could repeatedly divide without being depleted of PMPs. However, in the alternative view, if *de novo* peroxisome biogenesis occurs only when pre-existing peroxisomes are absent (van der Zand et al., 2010) and the *de novo* pathway serves only as a back-up process that is not utilized in cells constantly, it would have been eliminated from the system. In support of their postulate, they recently reported observations where pre-peroxisomal vesicular carriers (ppVs) carrying PMPs from the ER fuse only with each other but never with the pre-existing peroxisomes (van der Zand et al., 2012). This has challenged the role of ER in the maintenance of the pre-existing peroxisome population (Motley and Hettema, 2007).

## Conflicting reports on the role of ER in mammalian peroxisome biogenesis

In yeast, in spite of certain differences among various groups regarding the extent to which the ER contributes to peroxisome biogenesis, it is now widely accepted as a source of PMPs and membrane (including lipids) during *de novo* biogenesis of peroxisomes (Tabak et al., 2013; Theodoulou et al., 2013). However, such a consensus is not yet attained in mammalian systems. Several experimental setups that proved the involvement of ER in peroxisome biogenesis in yeast have yielded inconclusive results in mammalian cells. Firstly, while BFA did affect peroxisome biogenesis in yeast cells, it had no effect with mammalian cells (Salomons et al., 1997; Mullen et al., 1999; South and Gould, 1999; Voorn-Brouwer et al., 2001). In addition, there are inconsistent reports for the involvement of the components of the secretory pathway in peroxisome biogenesis in mammalian cells. Passreiter et al. reported the involvement of ARF and coatomer complex in peroxisome biogenesis in mammalian cells (Passreiter et al., 1998). This was contradicted in further reports showing that neither COPI, ARF1, or SAR1 (South et al., 2000; Voorn-Brouwer et al., 2001), nor Sec61 (South et al., 2001), are involved in peroxisome biogenesis in mammalian cells. Secondly, unlike yeast, mammalian cells with non-functional mutant of Pex3 or Pex19 or their knockdowns did not cause the ER accumulation of PMPs (South et al., 2000; Fang et al., 2004; Hunt and Trelease, 2004; Jones et al., 2004). In addition, several import assays were established in the mammalian cells for the *in vitro* import of PMPs (including TA proteins) to the peroxisomes directly from the cytosol (Diestelkotter, 1993; Imanaka et al., 1996; Matsuzono and Fujiki, 2006; Matsuzaki and Fujiki, 2008; Yagita et al., 2013).

Although, there are few independent reports suggesting a direct involvement of the ER in mammalian peroxisome biogenesis, a renewed focus on the contribution of ER in mammalian peroxisome biogenesis came when Gueze et al. convincingly showed the association of the ER with peroxisomes using advanced electron microscopy and three-dimensional image reconstruction in mouse dendritic cells (Geuze et al., 2003; Tabak et al., 2003), as proposed earlier(Rhodin, 1954; Ohno and Fujii, 1990). Using Immuno-gold labeling of mouse dendritic cells, it was found that lamellar structures enriched in Pex14 and PMP70 connected to the ER as its sub-domain (termed “specialized ER”), which is remarkably different from the rough ER in that it is less enriched with typical luminal ER markers, like PDI and calreticulin, and is devoid of attached ribosomes. Using 3-D reconstructions, they found that mature peroxisomes were also associated with similar lamellar structures that were not connected to, but sometimes showed membrane continuities with, the ER (Tabak et al., 2003). These structures might be similar to the “stalks” through which peroxisomes are connected with the ER (Rhodin, 1954).

More recently Kim et al. provided further evidence for the involvement of the ER in peroxisome biogenesis in mammalian cells (Kim et al., 2006). Using a photo-activatable, GFP-tagged Pex16, they showed that it is routed through the ER to the peroxisomes. They also demonstrated that *de novo* peroxisomes biogenesis contributes significantly more toward the total cellular pool of peroxisomes compared to the peroxisome population arising through growth and division (fission). Furthermore, they showed that Pex16 is first incorporated into the ER, which further recruits Pex3 and other PMPs to the membrane. This eventually leads to the differentiation of a “peroxisome-like” domain in the ER similar to those observed in mouse dendritic cells (Geuze et al., 2003). These “specialized ER” domains can detach from the ER to form peroxisomes *de novo* through a fission event. However, Pex3 and Pex16 were not in the ER when BFA was used to inhibit ER-mediated vesicle transport (Voorn-Brouwer et al., 2001). Nonetheless, they never tested the localization of these PMPs in *pex3*Δ or *pex19*Δ mutants. Kim et al. cited unpublished data where they observed a redistribution of Pex16 to the peroxisomes when cells were treated with BFA (Kim et al., 2006). Additionally, there are distinct mechanisms reported for the secretion of various proteins from the ER that do not require the COPI or COPII machinery and are thus insensitive to BFA (such as fibroblast growth factor, interleukin-1b, HIV-tat, galectin-3, thioredoxin) (Nickel, 2010). A further indication for an involvement of the ER in the biogenesis of mammalian peroxisomes came when Sec16B, a protein that defines ER exit sites, was overexpressed in HeLa cells (Yonekawa et al., 2011). It was found that Pex3, Pex16 along with Sec16B were redistributed and colocalized to the entire ER. However, a knockdown of Sec16B caused the ER retention of Pex16 and a suppression of Pex3 expression with a prominent effect on peroxisome morphology. Perhaps Sec16B recruits essential coat components to the pre-peroxisomal compartment at the ER for budding. Together, these results support the view that peroxisomes are also formed *de novo* from the ER in mammalian cells, thus creating a unified theme for peroxisome biogenesis with yeast and plants.

## Pre-peroxisomal intermediates and compartments

Several studies in mammalian and yeast cells have identified a peroxisomal pre-compartment in the ER (Geuze et al., 2003; Hoepfner et al., 2005; Tam et al., 2005), as well as various transitional precursors (Titorenko et al., 2000; Lam et al., 2010; Agrawal et al., 2011; van der Zand et al., 2012) that eventually mature into import-competent peroxisomes. In yeast, Pex3 is sorted first to the ER, where it further recruits other PMPs and transforms the site into a distinct compartment, often referred to as the “pre-peroxisomal ER (pER)” (Agrawal et al., 2011) or the “specialized ER” in mammalian cells (Geuze et al., 2003). In *S. cerevisiae*, these compartments are seen as one or two bright dots on the ER when a fluorescently tagged Pex3 is reintroduced in *pex3*Δ cells (Hoepfner et al., 2005; Tam et al., 2005; Agrawal et al., 2011). With time, a transient co-localization of Pex19 is seen (Hoepfner et al., 2005) which could be facilitating the budding process (Lam et al., 2010; Agrawal et al., 2011). However, in mammalian cells, Pex16 was first sorted to the “specialized-ER.” Pex16 is shown to be the anchoring receptor for Pex3 in the peroxisomal membrane, which further recruits Pex19 and other PMPs (Geuze et al., 2003; Tam et al., 2005; Kim et al., 2006; Schmidt et al., 2012). A localization of Pex19 to these structures on the ER is not yet detected. Interestingly, as mentioned before, repression of Sec16B expression restricts the Pex16 to the ER. Perhaps a role for Pex19 in recruiting Sec16B to these sites is an interesting possibility. Nonetheless, the mechanism that sorts PMPs after their import into the ER to the pER is still unknown although distinct signals have recently been found in yeast Pex3 for ER and pER sorting (Fakieh et al., 2013) (Figure 1).

In view of the observation that newly-synthesized Pex3 and other PMPs are trafficked through the ER to form mature peroxisomes, it was expected that the PMPs exit the ER in vesicular carriers. Previously, Titorenko and Rachubinki reported an extensive biochemical and morphological characterization of a multistep peroxisome maturation pathway in *Y. lipolitica* (Titorenko et al., 2000). Sucrose density gradient analysis identified five distinct precursor populations, each containing Pex2 and Pex16, but differing in their matrix enzyme compositions. It was found that a constant import of matrix enzymes and heterotypic fusion events result in the transformation of one vesicle type to another with higher density. However, it was unclear that whether they originated upon budding from the ER or through fission of pre-existing peroxisomes.

Recently, two independent studies performed in *S. cerevisiae* and *P. pastoris* identified vesicular carriers that bud from the ER carrying PMPs (Lam et al., 2010; Agrawal et al., 2011). In *P. pastoris*, we showed that budded vesicles carried two different PMPs, Pex3, and Pex11, co-packaged together in the same vesicle in an ATP-dependent manner. Importantly, the budding process required Pex19, but not Pex3 or other peroxins (Pex1, Pex5, Pex7, and Pex14). However, the budded vesicles detected in *pex3*Δ cells carried a very limited repertoire of PMPs and lacked matrix proteins. Nonetheless, this was a surprising result since Pex3 was believed to be critical for docking of Pex19 at the ER. It further raised the possibility that Pex19 could dock with other peroxins at the ER membrane for initiating the budding process, while Pex3 might be critical for maturation of ppVs into import competent peroxisomes. More recently, van der Zand et al. showed that the pre-peroxisomal vesicles that bud from the ER are of at least two types, each carrying subcomponents of the peroxisomal translocon complex (van der Zand et al., 2012). Import competent peroxisomes were formed with heterotypic fusion of these vesicles, which fuse only with each other, but not with the pre-existing mature peroxisomes. Segregation of the peroxisomal translocon complex components into distinct compartments also suggested a way to keep the ER from importing the peroxisomal matrix enzymes. However, the biochemical requirements for the budding of these heterogeneous vesicles were not identified, leaving the possibility for the need for non-overlapping components specific for each type of vesicle (Figure 1).

## Revisiting the role of Pex19 in peroxisome biogenesis

Several studies during the last decade have bought a paradigm shift in our understanding of the mechanistic role of Pex19 in peroxisome biogenesis. Pex19 has been ascribed multiple roles in peroxisome biogenesis pathway including the PMP receptor, the budding of ppVs, peroxisome division, as well as inheritance. Most importantly, Pex19 is considered as a PMP-chaperone and a shuttling receptor. Because several PMPs contain one or more mPTS for binding Pex19 and these PMPs are unstable or aggregate in the absence of Pex19, the Pex19 protein is considered essential for binding and stabilizing PMPs in the cytosol (chaperone like activity) (Sacksteder et al., 2000; Jones et al., 2004; Shibata et al., 2004; Kashiwayama et al., 2005). Moreover, it is presumed that Pex19 delivers the bound PMPs to the peroxisome by docking with Pex3 at the peroxisomal membrane (Fang et al., 2004; Rottensteiner et al., 2004; Hoepfner et al., 2005; Matsuzono and Fujiki, 2006). The role of Pex19 is often extended to insertion of PMPs into peroxisomal membrane as well (Sacksteder et al., 2000; Jones et al., 2004), where it is presumed to incorporate PMP. Following this step, Pex19 is recycled to the cytosol for the next round of insertion (Schmidt et al., 2012).

Most of the studies cited above are performed in mammalian cells, where growth and division is the more prevalent model. In yeast, the role of Pex19 in the direct insertion of PMPs into the peroxisomal membrane has been actively debated (Snyder et al., 2000; Hoepfner et al., 2005; van der Zand et al., 2010) with an alternative emerging role for Pex19 in the budding of ppVs (Lam et al., 2010; Agrawal et al., 2011). Presumably, Pex19 docks on Pex3 through its Pex3-binding or PMP-binding domains to recruit other components of the budding machinery (still unidentified). However, since components of the conventional secretory pathway are not involved in the budding process, Pex19 could be speculated to assemble a machinery similar to that for peroxisome division at the pER. The action of such membrane fission machinery, in concert with the Pex11-like proteins that cause membrane tubulation (Opalinski et al.), could result in the budding of ER-derived ppVs. This is a conceivable scenario since Pex19 interacts with several proteins involved in the peroxisome division process including Vps1, Fis1, Pex11, and Pex25 (Rottensteiner et al., 2004; Vizeacoumar et al., 2006; Delille and Schrader, 2008; Tarassov et al., 2008; Rucktäschel et al., 2009). This idea remains to be tested experimentally.

## Converging pathways

Our view of peroxisome biogenesis is now being transformed by multiple studies either supporting the *de novo* pathway for peroxisome biogenesis (van der Zand et al., 2010, 2012) or by those depicting peroxisomes as autonomous organelles that replenish themselves by growth and division (Lazarow, 2003; Nagotu et al., 2008). However, a handful of studies have suggested that both pathways might operate simultaneously (Kim et al., 2006; Huber et al., 2011), or could be conditionally segregated (Motley and Hettema, 2007). Since both pathways eventually lead to peroxisome biogenesis, it is natural to see them as two sides of the same coin. Evidently, when key components of one pathway are blocked, the other pathway takes charge to replenish the organelle supply, but when key components of both the pathways are blocked, the lack of peroxisome biogenesis is evident (Huber et al., 2011; Saraya et al., 2011). In addition, studies with an impaired division or inheritance machinery reveal a slowed biogenesis process (Kim et al., 2006; Motley and Hettema, 2007; Joshi et al., 2012). Nonetheless, cells form functional peroxisomes presumably through the *de novo* pathway. This could also suggest that both pathways need to operate simultaneously to make the organelle regeneration and maintenance kinetically efficient. If true, the alternative models of peroxisome generation described herein might not be mutually exclusive, but rather redundant mechanisms evolved for infallible organelle regeneration. Cross-talk between the two pathways might be essential for achieving dynamic peroxisome homeostasis (Figure 1). These remain as interesting topics for further exploration.

### Conflict of interest statement

The authors declare that the research was conducted in the absence of any commercial or financial relationships that could be construed as a potential conflict of interest.

## Abbreviations

pER: pre-peroxisomal ER
ppVs: pre-peroxisomal vesicles
PMP: peroxisomal membrane protein.
